# Effects of acoustic stimulation intensity on air-conducted vestibular evoked myogenic potential in children

**DOI:** 10.3389/fneur.2022.996246

**Published:** 2022-10-11

**Authors:** Qianwen Xiao, Qin Zhang, Qiong Wu, Jiali Shen, Lu Wang, Yanfei Chen, Jingrong Lv, Jun Yang, Yulian Jin, Qing Zhang

**Affiliations:** ^1^Department of Otorhinolaryngology-Head and Neck Surgery, Xinhua Hospital, Shanghai Jiaotong University School of Medicine, Shanghai, China; ^2^Shanghai Jiaotong University School of Medicine Ear Institute, Shanghai, China; ^3^Shanghai Key Laboratory of Translational Medicine on Ear and Nose Diseases, Shanghai, China; ^4^Department of Otolaryngology, Second Affiliated Hospital of Xi'an Jiaotong University, Xi'an, China

**Keywords:** acoustic stimulation intensity, air-conducted sound, ocular vestibular evoked myogenic potential, cervical vestibular evoked myogenic potential, children

## Abstract

**Objective:**

To investigate the effects of acoustic stimulation intensity on ocular and cervical vestibular evoked myogenic potential (oVEMP and cVEMP) responses elicited by air-conducted sound (ACS) in healthy children.

**Methods:**

Thirteen healthy children aged 4–10 years and 20 healthy adults aged 20-40 years with normal hearing and tympanometry were enrolled in this study. All subjects received oVEMP and cVEMP tests under different acoustic stimulation intensities (131, 126, 121, 116, 111 and 106 dB SPL). Mean n1 latency, p1 latency, interpeak latency, amplitude and response rate were investigated and analyzed.

**Results:**

As the acoustic stimulation intensity decreased, for oVEMP, the response rate of children decreased from 100% (131, 126 and 121 dB SPL) to 57.69% (116 dB SPL), 26.92% (111 dB SPL) and 11.54% (106 dB SPL). The response rate of adults decreased from 100% (131 and 126 dB SPL) to 95% (121 dB SPL), 55% (116 dB SPL), 12.5% (111 dB SPL) and 2.5% (106 dB SPL). There were lower n1 latency, p1 latency and higher amplitude in children when comparing by acoustic stimulation intensities (*p* < 0.05). Regarding cVEMP, the response rate of children decreased from 100% (131, 126 and 121 dB SPL) to 88.46% (116 dB SPL), 53.85% (111 dB SPL) and 26.92% (106 dB SPL). The response rate of adults decreased from 100% (131 and 126 dB SPL) to 95% (121 dB SPL), 85% (116 dB SPL), 37.5% (111 dB SPL) and 7.5% (106 dB SPL). A statistically significant difference was found in amplitude at different acoustic stimulation intensities in both children and adults (*p* < 0.05). When stimulated by 131 dB SPL acoustic stimulation, there were lower n1 latency, p1 latency and higher amplitude in children in oVEMP and cVEMP compared with adults (*p* < 0.05).

**Conclusion:**

The response rate and amplitude of oVEMP and cVEMP in children and adults presented significant differences with a decrease in acoustic stimulation intensity. In this study, using 121 dB SPL for children and 126 dB SPL for adults during VEMP test could be regarded as safer stimulation intensities and thus reduced sound exposure.

## Introduction

Vestibular evoked myogenic potential (VEMP) has been utilized in neuro-otology clinics as a test for evaluation of otolith function and vestibular nerves (1, 2). It is elicited by modulated electromyographic signals either from the inferior oblique muscle for the ocular VEMP (oVEMP) or the sternocleidomastoid muscle for the cervical VEMP (cVEMP) (3). There are usually three types of stimuli eliciting VEMPs, including air-conducted sound (ACS), bone-conducted vibration (BCV) and galvanic vestibular stimulation (GVS), among which ACS has been regarded as the primary and widely used stimulus (4, 5). Previous studies have demonstrated that, when evoked by ACS, the oVEMP could evaluate utricle function and the crossed vestibulo-ocular reflex (VOR) and the cVEMP could evaluate saccular function and the vestibulo-collic reflex (VCR) pathway (2, 4, 6, 7).

Vestibular loss often resulted in delayed motor development and reduced quality of life in children with normal hearing (8). The prevalence of childhood balance disorders is uncertain and mainly depends on the method of data collection. It is estimated that about 0.45% children aged from newborns to 18 years are diagnosed with balance disturbances (9). However, the prevalence may be even higher and the underestimation can be attributed to difficulties in describing vertigo, obtaining detailed medical history and establishing clear diagnosis (8). Therefore, vestibular loss in children is in need of attention, and clinicians are supposed to search for a valid and reliable tool to increase the diagnostic rate in children.

The oVEMP and cVEMP tests *via* ACS are objective, non-invasive and safe to perform in children as long as a safe acoustic stimulation is maintained (10, 11). The test has been widely used in adults and the normal values have been identified (12). Previous study had only focused on the effects of simple acoustic stimulation intensity on VEMPs. However, few studies on the sets of normative data in children have been reported, especially the investigation on the effects of different acoustic stimulation intensity on ACS-VEMPs in children. Thus, more researches are necessary and critical to determine the appropriate acoustic stimulation intensity in the tests. The aim of this study is to investigate different acoustic stimulation intensities on VEMPs elicited by ACS in healthy children.

## Materials and methods

### Subjects

Thirteen healthy children (6 males and 7 females, aged from 4 to 10 years, mean 7.23 ± 2.01 years) and 20 healthy adults (9 males and 11 females, aged from 20 to 40 years, mean 24.95 ± 5.16 years) were enrolled in this study. All subjects had no history of any ear disorders and vestibular disorders, and were further checked with pure tone audiometry, acoustical immittance and otoscope tests. Each subject underwent oVEMP and cVEMP elicited by ACS. This study was approved by the institutional review board of the Xinhua Hospital of Shanghai Jiaotong University School of Medicine. Each child's parent and each adult signed the informed consent to take part in the study.

### Equipment and recordings

A sound-proof and comfortable examination room was employed for tests. The electromyographic signals were amplified through the ICS Chartr EP 200 Evoked Potential System (Otometrics, Denmark) for further analysis.

ACS with 500 Hz short tone burst (rise/fall time = 1ms, plateau time = 2ms) was delivered through the inserted earphone. The band-pass filter was set at 1–1000 Hz, and the responses to 50 stimuli were averaged twice. The stimulation rate was 5 Hz, and the analysis window of each response was 50 ms. The initial acoustic stimulus used was a short tone burst, with an intensity of 131 dB SPL. The stimulation intensity was then decreased in steps of 5 dB SPL until no oVEMP or cVEMP were present. A clear and repeatable biphasic waveform comprised of peaks n1 and p1 was considered positive response, and unrepeatable biphasic waveform was considered no response. The length of time between 0 ms and the peak n1 or p1 was regarded as n1 latency or p1 latency, respectively. The duration between peaks n1 and p1 was recorded as interpeak latency, which includes n1-p1 latency and p1-n1 latency. We regarded the vertical distance of voltage between peaks n1 and p1 as the amplitude.

### oVEMP test

Each subject was in the supine position during the test. Before attaching electrodes, the skin of all subjects should be cleaned with abrasive paste. Two active electrodes were positioned around 1 cm below the center of the two lower eyelids. Two reference electrodes were placed around 1–2 cm below the two active electrodes, and the ground electrode was placed on the middle of the forehead. The electrode impedance was kept below 5 kΩ. Each subject was asked to look upward at a small fixed target above 1 m from the eyes when hearing the sound through the inserted earphone (13). Response rate, n1 and p1 latencies, n1-p1 latency and amplitude were measured under different acoustic stimulation intensities.

### cVEMP test

Each subject was in the supine position during the test. Before attaching electrodes, the skin of all subjects was cleaned with abrasive paste. Two active electrodes were placed on the middle and upper third of the sternocleidomastoid (SCM) muscle, and the two reference electrodes were positioned on jugular notch. The ground electrode was placed on the middle of the forehead. The electrode impedance was kept below 5 kΩ. Each subject was instructed to raise his/her head off the pillow in order to increase the tension of the SCM when the sound was presented through the inserted earphone (14). Response rate, p1 and n1 latencies, p1-n1 latency and amplitude were measured under different acoustic stimulation intensities.

### Statistical methods

Data were analyzed using IBM SPSS Statistics 26.0.0. Kruskal-Wallis test was used for comparisons of n1 latency, p1 latency, interpeak latency and amplitude of oVEMP or cVEMP among different acoustic stimulation intensities. All data were expressed as mean ± standard deviation. A significance of *p* < 0.05 is considered significant.

## Results

### Acoustic stimulation intensity impacts on ACS-oVEMP in children

**Figure 1:**
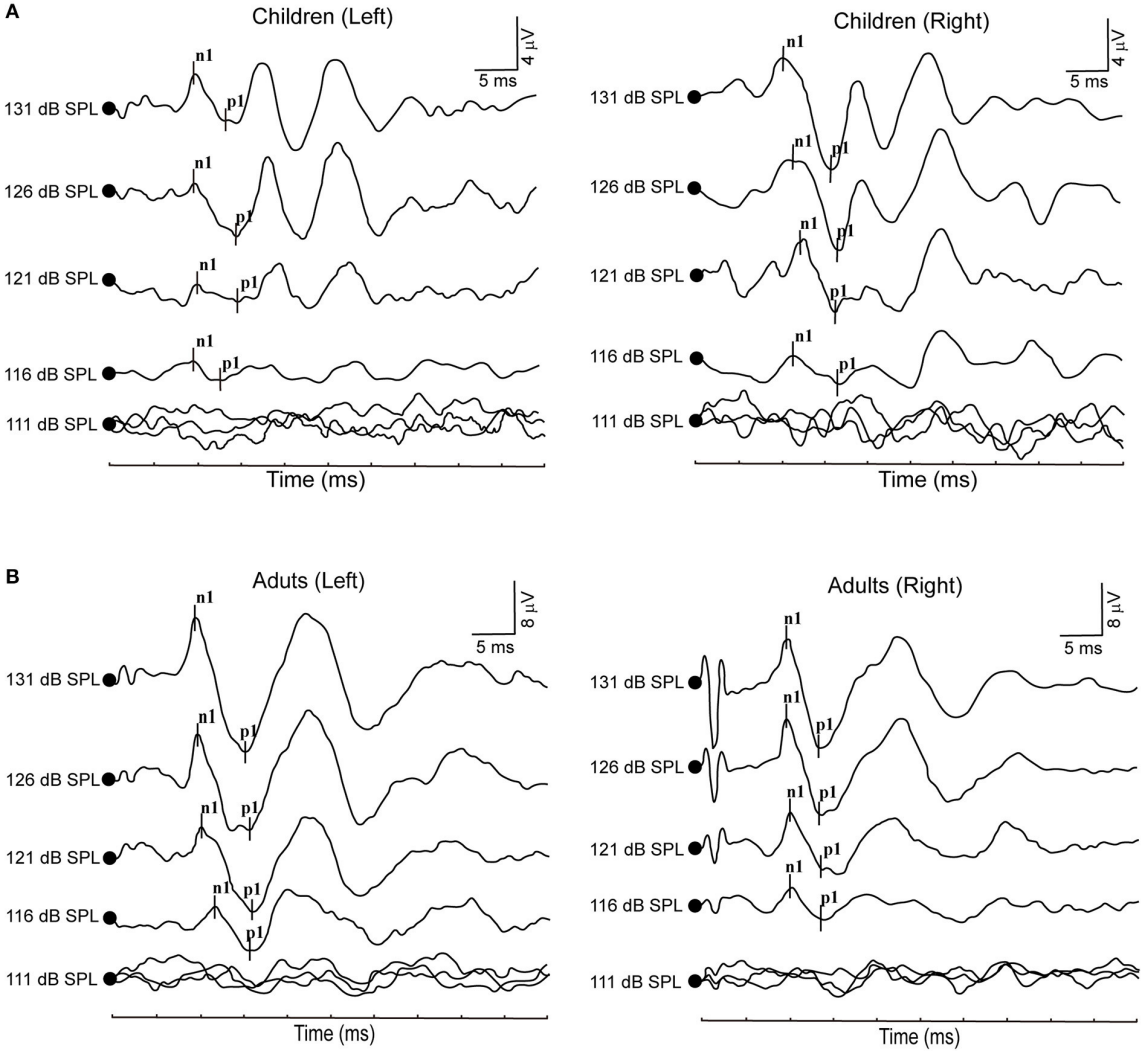
**(A)** Clear oVEMP waveform in a child in response to ACS stimuli. **(B)** Clear oVEMP waveform in an adult in response to ACS stimuli.

All healthy children completed ACS-oVEMP test following different acoustic stimulation intensities, which included 131, 126, 121, 116, 111 and 106 dB SPL (Table 1, Figure 1A). Regarding oVEMP, the response rates were 100% (26/26) under 131, 126 and 121 dB SPL acoustic stimulations. However, the response rate decreased gradually (57.69, 26.92 and 11.54%, respectively) under 116, 111 and 106 dB SPL acoustic stimulations. As acoustic stimulation intensity decreased, the mean n1 latencies increased (9.97 ± 0.75 ms, 10.29 ± 0.69 ms, 10.56 ± 1.01 ms, 10.78 ± 0.86 ms, 11.88 ± 0.75 ms and 11.96 ± 0.18 ms, respectively) and the mean p1 latencies increased (14.41 ± 1.18 ms, 14.89 ± 0.93 ms, 15.16 ± 1.09 ms, 15.37 ± 0.99 ms, 15.70 ± 0.93 ms and 16.71 ± 0.30 ms, respectively) and the mean amplitudes decreased (8.32 ± 5.71 μV, 6.53 ± 3.57 μV, 3.99 ± 2.70 μV, 2.90 ± 1.44 μV, 2.65 ± 0.86 μV and 2.37 ± 1.39 μV, respectively). Comparisons of parameters showed prolonged latencies of n1 (*p* < 0.0001) and p1 (*p* = 0.010) and decreased amplitude (*p* < 0.0001) significantly. Whereas, no significant difference was observed in the n1-p1 latency (*p* = 0.418).

**Table 1:** The ACS-oVEMP with decreasing acoustic stimulation intensity in children.

**Intensity (dB SPL)**	**N (ears)**	**Response rate**	**n1 latency (ms)**	**p1 latency (ms)**	**Interpeak latency (ms)**	**Amplitude (μV)**
131	26	100%	9.97 ± 0.75	14.41 ± 1.18	4.45 ± 1.10	8.32 ± 5.71
126	26	100%	10.29 ± 0.69	14.89 ± 0.93	4.60 ± 0.83	6.53 ± 3.57
121	26	100%	10.56 ± 1.01	15.16 ± 1.09	4.61 ± 1.17	3.99 ± 2.70
116	15	57.69%	10.78 ± 0.86	15.37 ± 0.99	4.59 ± 0.92	2.90 ± 1.44
111	7	26.92%	11.88 ± 0.75	15.70 ± 0.93	3.82 ± 0.58	2.65 ± 0.86
106	3	11.54%	11.96 ± 0.18	16.71 ± 0.30	4.75 ± 0.11	2.37 ± 1.39
Kruskal-Wallis test			*p* < 0.0001	*p =* 0.010	*p =* 0.418	*p* < 0.0001

Data are expressed as mean ± SD.

### Acoustic stimulation intensity impacts on ACS-cVEMP in children

**Figure 2:**
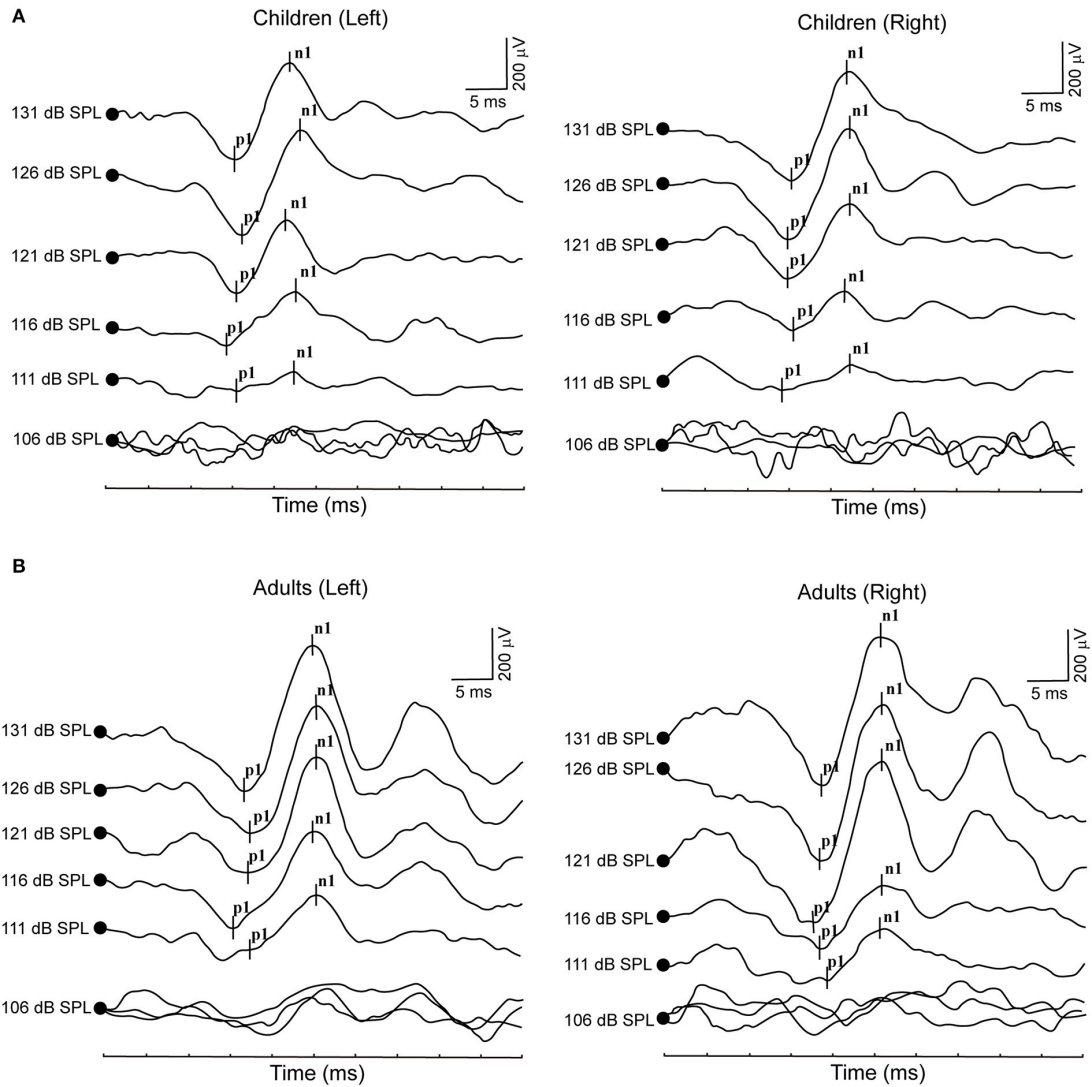
**(A)** Clear cVEMP waveform in a child in response to ACS stimuli. **(B)** Clear cVEMP waveform in an adult in response to ACS stimuli.

All healthy children completed ACS-cVEMP test under 131, 126, 121, 116, 111 and 106 dB SPL acoustic stimulation intensities (Table 2, Figure 2A). Regarding cVEMP, the response rates were 100% (26/26) under 131, 126 and 121 dB SPL acoustic stimulations. Whereas, the response rate decreased from 88.46, 53.85 to 26.92% under 116, 111 and 106 dB SPL acoustic stimulations, respectively. With the decrease of acoustic stimulation intensity, the mean amplitudes were 369.60 ± 177.90 μV, 402.80 ± 163.90 μV, 271.60 ± 155.60 μV, 228.70 ± 118.00 μV, 177.80 ± 96.56 μV and 150.80 ± 81.22 μV, indicating decreasing acoustic stimulation intensity was accompanied by a significant decrease of amplitude (*p* < 0.0001). However, statistically significant differences were not found in terms of p1 latency (*p* = 0.310), n1 latency (*p* = 0.542) and p1-n1 latency (*p* = 0.826).

**Table 2:** The ACS-cVEMP with decreasing acoustic stimulation intensity in children.

**Intensity (dB SPL)**	**N (ears)**	**Response rate**	**n1 latency (ms)**	**p1 latency (ms)**	**Interpeak latency (ms)**	**Amplitude (μV)**
131	26	100%	21.45 ± 1.58	14.96 ± 1.08	6.52 ± 1.00	369.60 ± 177.90
126	26	100%	22.15 ± 1.76	15.67 ± 1.27	6.49 ± 1.01	402.80 ± 163.90
121	26	100%	22.08 ± 1.89	15.47 ± 1.34	6.61 ± 1.35	271.60 ± 155.60
116	23	88.46%	21.66 ± 1.48	15.40 ± 1.29	6.27 ± 1.12	228.70 ± 118.00
111	14	53.85%	21.70 ± 1.44	14.99 ± 0.77	6.69 ± 1.11	177.80 ± 96.56
106	7	26.92%	21.00 ± 1.14	14.82 ± 0.92	6.18 ± 1.40	150.80 ± 81.22
Kruskal-Wallis test			*p =* 0.542	*p =* 0.310	*p =* 0.826	*p* < 0.0001

Data are expressed as mean ± SD.

### Acoustic stimulation intensity impacts on ACS-oVEMP in adults

All healthy adults presented ACS-oVEMP test induced by 131, 126, 121, 116, 111 and 106 dB SPL acoustic stimulation intensities (Table 3, Figure 1B). The response rates were 100% (40/40) when simulated by 131 and 126 dB SPL acoustic stimulations. However, the response rate decreased from 95, 55, 12.5% to 2.5% under 121, 116, 111 and 106 dB SPL acoustic stimulations, respectively. As the acoustic stimulation intensity decreased, the mean n1 latencies were 10.26 ± 0.68 ms, 10.43 ± 0.78 ms, 10.74 ± 0.84 ms, 11.38 ± 1.09 ms, 12.12 ± 1.27 ms and 12.58 ± 0.00 ms, and the mean p1 latencies were 14.76 ± 1.31 ms, 14.94 ± 1.40 ms, 15.25 ± 1.17 ms, 15.79 ± 0.98 ms, 16.00 ± 0.94 ms and 16.00 ± 0.00 ms, and the mean amplitudes were 7.29 ± 3.60 μV, 5.82 ± 3.38 μV, 3.85 ± 2.16 μV, 3.52 ± 2.03 μV, 3.23 ± 0.67 μV and 3.21 ± 0.00 μV. Comparisons of parameters revealed that there were significant differences in the n1 latency (*p* < 0.0001), p1 latency (*p* = 0.015) and amplitude (*p* < 0.0001), but not in the n1-p1 latency (*p* = 0.690).

**Table 3:** The ACS-oVEMP with decreasing acoustic stimulation intensity in adults.

**Intensity (dB SPL)**	**N (ears)**	**Response rate**	**n1 latency (ms)**	**p1 latency (ms)**	**Interpeak latency (ms)**	**Amplitude (μV)**
131	40	100%	10.26 ± 0.68	14.76 ± 1.31	4.50 ± 1.07	7.29 ± 3.60
126	40	100%	10.43 ± 0.78	14.94 ± 1.40	4.51 ± 1.19	5.82 ± 3.38
121	38	95%	10.74 ± 0.84	15.25 ± 1.17	4.51 ± 1.13	3.85 ± 2.16
116	22	55%	11.38 ± 1.09	15.79 ± 0.98	4.55 ± 1.03	3.52 ± 2.03
111	5	12.50%	12.12 ± 1.27	16.00 ± 0.94	3.88 ± 0.83	3.23 ± 0.67
106	1	2.50%	12.58 ± 0.00	16.00 ± 0.00	3.42 ± 0.00	3.21 ± 0.00
Kruskal-Wallis test			*p* < 0.0001	*p =* 0.015	*p =* 0.690	*p* < 0.0001

Data are expressed as mean ± SD.

### Acoustic stimulation intensity impacts on ACS-cVEMP in adults

All healthy adults completed ACS-cVEMP test induced by 131, 126, 121, 116, 111 and 106 dB SPL acoustic stimulation intensities (Table 4, Figure 2B). The response rates were 100% (40/40) under 131 and 126 dB SPL acoustic stimulations. Whereas, the response rate gradually decreased (95, 85, 37.5 and 7.5%, respectively) when induced by 121, 116, 111 and 106 dB SPL acoustic stimulations. With the decrease of acoustic stimulation intensity, the mean amplitudes were 279.50 ± 151.20 μV, 253.80 ± 128.50 μV, 230.80 ± 110.90 μV, 179.10 ± 80.38 μV, 155.30 ± 57.60 μV and 142.40 ± 44.49 μV, respectively. Although there was a significant difference in the amplitude (*p* = 0.002), no statistically significant differences were not found in terms of p1 latency (*p* = 0.277), n1 latency (*p* = 0.974) and p1-n1 latency (*p* = 0.190).

**Table 4:** The ACS-cVEMP with decreasing acoustic stimulation intensity in adults.

**Intensity (dB SPL)**	**N (ears)**	**Response rate**	**n1 latency (ms)**	**p1 latency (ms)**	**Interpeak latency (ms)**	**Amplitude (μV)**
131	40	100%	24.97 ± 1.87	17.15 ± 1.94	7.82 ± 1.37	279.50 ± 151.20
126	40	100%	25.08 ± 2.10	17.30 ± 2.14	7.78 ± 1.37	253.80 ± 128.50
121	38	95%	25.23 ± 2.13	17.82 ± 2.34	7.39 ± 1.41	230.80 ± 110.90
116	34	85%	25.02 ± 2.31	17.64 ± 2.33	7.39 ± 1.95	179.10 ± 80.38
111	15	37.50%	24.90 ± 2.32	18.27 ± 2.45	6.63 ± 1.67	155.30 ± 57.60
106	3	7.50%	24.64 ± 1.76	17.25 ± 1.08	7.39 ± 1.06	142.40 ± 44.49
Kruskal-Wallis test			*p =* 0.974	*p =* 0.277	*p =* 0.190	*p =* 0.002

Data are expressed as mean ± SD.

### oVEMP and cVEMP: Children vs. adults

All children and adults presented VEMPs following 131 dB SPL acoustic stimulation (Table 5). Regarding oVEMP, mean n1 latency, p1 latency, n1-p1 latency and amplitude for children were 9.97 ± 0.75 ms, 14.41 ± 1.18 ms, 4.45 ± 1.10 ms and 8.32 ± 5.71 μV, respectively, and 10.26 ± 0.68 ms, 14.76 ± 1.31 ms, 4.50 ± 1.07 ms and 7.29 ± 3.60 μV for adults, respectively, indicating that latencies were shorter in children than that in adults. There was a significant difference in the n1 latency between children and adults (*p* = 0.007), but not in the p1 latency (*p* = 0.288), n1-p1 latency (*p* = 0.752) and amplitude (*p* = 0.807). For cVEMP, mean p1 latency, n1 latency, p1-n1 latency and amplitude were 14.96 ± 1.08 ms, 21.45 ± 1.58 ms, 6.52 ± 1.00 ms and 369.6 ± 177.9 μV for children, while 17.15 ± 1.94 ms, 24.97 ± 1.87 ms, 7.82 ± 1.37 ms and 279.5 ± 151.2 μV for adults, respectively, indicating that children had shorter latencies and lower amplitudes than that in adults. A significant difference existed between children and adults in terms of p1 latency (*p* < 0.0001), n1 latency (*p* < 0.0001), p1-n1 latency (*p* < 0.0001) and amplitude (*p* = 0.021).

**Table 5:** oVEMP and cVEMP under 131 dB SPL stimulation in children vs. adults.

	**oVEMP**		***p***	**cVEMP**		***p***
	**Children**	**Adults**		**Children**	**Adults**	
n1 latency (ms)	9.97, 0.75	10.26, 0.68	0.007	21.45, 1.58	24.97, 1.87	<0.0001
p1 latency (ms)	14.41, 1.18	14.76, 1.31	0.288	14.96, 1.08	17.15, 1.94	<0.0001
Interpeak latency (ms)	4.45, 1.10	4.50, 1.07	0.752	6.52, 1.00	7.82, 1.37	<0.0001
Amplitude (μV)	8.32, 5.71	7.29, 3.60	0.807	369.6, 177.9	279.5, 151.2	0.021

Data are expressed as mean ± SD.

## Discussion

VEMPs have been widely utilized in children suspected with peripheral vestibular disorders due to early maturation of the crossed VOR and the VCR (15). Though there are several types of stimuli eliciting VEMPs, ACS is presumed to be the most commonly used in the clinical setting (16). To our knowledge, the risk for ACS-VEMPs test in children and adults is the increased sound exposure on account of the number of tests required in order to obtain a response. Previous studies have observed adverse effects on cochlear function resulted from VEMPs test in adults, including sudden sensorineural hearing loss, decreased distortion product otoacoustic emission (DPOAE) amplitudes and other symptoms (17–19). Compared to the adults, there are few investigations concerning the effect of acoustic stimulation intensity on ACS-VEMPs in children. In the article, we therefore investigated the characteristics of ACS-VEMPs induced by different acoustic stimulation intensities in children for searching for an appropriate acoustic stimuli level and avoiding the potential risk of acoustic trauma associated with VEMPs test.

In the current study, our results revealed that the response rates were 100% when stimulated by 131, 126 and 121 dB SPL acoustic stimulations in children. Compared to children, the response rates of adults were 100% under 131 and 126 dB SPL acoustic stimulations. Based upon these results, 121 and 126 dB SPL were regarded as the appropriate initial acoustic stimulation intensity for VEMPs test in children and adults, respectively. This indicated that VEMPs stimuli for children may not need to be presented adopting adults stimulation levels. As reported by Rodriguez et al., children receive around 3 dB higher peak equivalent SPL in the ear in response to acoustic stimulation due to the smaller equivalent ear canal volumes (ECV) of children compared to adults. Therefore, a 120 dB SPL acoustic stimulation intensity is recommended for VEMPs test in children with ECV below 0.8 cm, which is similar to our results (20). In addition, we also found the amplitude significantly attenuated in both children and adults with the reduction in acoustic stimulation intensity, indicating a close relationship existed between acoustic stimulation intensity and the amplitude (21). Interestingly, oVEMP showed significantly prolonged n1 and p1 latencies with the decrease of acoustic stimulation intensity not only in children but also in adults. Taken together, our findings supported the notion that different acoustic stimulation intensities had significantly impacts on the n1 latency, p1 latency, amplitude of oVEMP and the amplitude of cVEMP in both children and adults.

On the other hand, we investigated the characteristics of VEMPs induced by 131 dB SPL acoustic stimulation between children and adults. Regarding oVEMP, since the conduction velocity increased with age to compensate for increasing brainstem circumference, Hsu et al. have demonstrated that significant differences in oVEMP parameters were not found between children and adults (22). Whereas, the current data revealed that children had shorter oVEMP n1 latencies compared to adults. This needs to be further verified through increasing the number of samples. Additionally, our results showed that cVEMP p1 and n1 latencies were significantly shorter in children under 131 dB SPL acoustic stimulation compared to adults, which may be ascribed to several factors consisting of VCR pathways development, neck length and head size in children (23). We detected the cVEMP amplitude for adults attenuated compared to that for children, which is different from previous view (24, 25). We speculated that different acoustic stimulation intensities and the increased number of trials resulted in fatigue of the sternocleidomastoid muscle.

Children may be at higher risk for noise-induced hearing loss from sound exposure. Previous studies in animal models demonstrated that young mice are more prone to neural degeneration through the cochlear when exposed to high acoustic stimulations compared to older mice (26). Though there are no available human data, the corresponding findings in mice made us aware of the importance of children's acoustic exposure from VEMPs stimulations. Apart from acoustic stimulation intensity, VEMPs response depends on frequency, rise/fall and plateau time and duration, and these parameters can affect the total sound pressure level (SPL) delivered to children's ears in ACS-VEMPs test (27). This study is dedicated to investigating the characteristics of acoustic stimulation intensity on ACS-VEMPs in healthy children. However, certain populations with some disorders in clinical practice, including tinnitus or hyperacusis, third-window phenomena and high susceptibility to noise-induced hearing loss, should also be taken into consideration to avoid potential acoustic trauma in VEMPs test (28). We could collect medical history, make hearing test and vestibular function examinations and do imaging test to exclude those diseases. Moreover, in this article, there are some limitations we should take into consideration. Since the children coordination is worse that of adults during VEMPs test, the sample size of children and age ranges were small, and EMG monitoring was not completed. Therefore, clinicians must be mindful of all factors associated with potential acoustic trauma, and further studies are needed to search for an appropriate acoustic stimulation intensity protocol to minimize the risk of unsafe sound exposure during VEMPs test in children.

## Conclusion

Findings from the study showed significant differences in the response rate and amplitude in VEMPs in both children and adults when stimulated by different acoustic stimulation intensities. We suggested that 121 and 126 dB SPL were considered as the appropriate initial acoustic stimulation intensity for VEMPs test in children and adults, respectively.

## Data availability statement

The original contributions presented in the study are included in the article/supplementary material, further inquiries can be directed to the corresponding authors.

## Ethics statement

The studies involving human participants were reviewed and approved by Xinhua Hospital of Shanghai Jiaotong University School of Medicine. Written informed consent to participate in this study was provided by the participants' legal guardian/next of kin.

## Author contributions

QX, QinZ, QW, JS, LW, and YC contributed to the data collection. QX wrote the manuscript. YJ, QingZ, JY, and JL provided the idea and edited the manuscript. All authors contributed to manuscript revision and approved the submitted version.

## Funding

This study was supported by the National Natural Science Foundation of China (No. 82171137, No. 81970891, and No. 81970876), The Key International Cooperation Project of Shanxi Province (Grant No. 2020-KWZ-019), Technology Project of Shanghai Science and Technology Commission (Grant No. 21S31900600), and Clinical Research Plan of SHDC (Grant No. SHDC2022CRD013).

## Conflict of interest

The authors declare that the research was conducted in the absence of any commercial or financial relationships that could be construed as a potential conflict of interest.

## Publisher's note

All claims expressed in this article are solely those of the authors and do not necessarily represent those of their affiliated organizations, or those of the publisher, the editors and the reviewers. Any product that may be evaluated in this article, or claim that may be made by its manufacturer, is not guaranteed or endorsed by the publisher.

## Abbreviations

VEMP: vestibular evoked myogenic potential
oVEMP: ocular VEMP
cVEMP: cervical VEMP
ACS: air-conducted stimulation
BCV: bone-conducted vibration
GVS: galvanic vestibular stimulation
VOR: vestibulo-ocular reflex
VCR: vestibulo-collic reflex
SCM: sternocleidomastoid muscle
DPOAE: distortion product otoacoustic emission
ECV: ear canal volume.

## References

[1] Taylor RL Welgampola MS Nham B Rosengren SM. Vestibular-evoked myogenic potential testing in vestibular localization and diagnosis. Semin Neurol. (2020) 40:18–32. 10.1055/s-0039-3402068 31935772

[2] Rosengren SM Colebatch JG Young AS Govender S Welgampola MS. Vestibular evoked myogenic potentials in practice: methods, pitfalls and clinical applications. Clin Neurophysiol Pract. (2019) 4:47–68. 10.1016/j.cnp.2019.01.005 30949613 PMC6430081

[3] Colebatch JG Rosengren SM Welgampola MS. Vestibular-evoked myogenic potentials. Handb Clin Neurol. (2016) 137:133–55. 10.1016/B978-0-444-63437-5.00010-8 27638068

[4] Curthoys IS A. critical review of the neurophysiological evidence underlying clinical vestibular testing using sound, vibration and galvanic stimuli. Clin Neurophysiol. (2010) 121:132–44. 10.1016/j.clinph.2009.09.027 19897412

[5] Curthoys IS Iwasaki S Chihara Y Ushio M McGarvie LA Burgess AM. The ocular vestibular-evoked myogenic potential to air-conducted sound; probable superior vestibular nerve origin. Clin Neurophysiol. (2011) 122:611–6. 10.1016/j.clinph.2010.07.018 20709596

[6] Curthoys IS Grant JW Burgess AM Pastras CJ Brown DJ Manzari L. otolithic receptor mechanisms for vestibular-evoked myogenic potentials: a review. Front Neurol. (2018) 9:366. 10.3389/fneur.2018.00366 29887827 PMC5980960

[7] Murofushi T Wakayama K. Chihara Y. oVEMP to air-conducted tones reflects functions of different vestibular populations from cVEMP?. Eur Arch Otorhinolaryngol. (2010) 267:995–6. 10.1007/s00405-010-1246-7 20376469

[8] Lee JD Kim CH Hong SM. Prevalence of vestibular and balance disorders in children and adolescents according to age: a multi-center study. Int J Pediatr Otorhinolaryngol. (2017) 94:36–9. 10.1016/j.ijporl.2017.01.012 28167008

[9] O'Reilly RC Morlet T Nicholas BD. Prevalence of vestibular and balance disorders in children. Otol Neurotol. (2010) 31:1441–4. 10.1097/MAO.0b013e3181f20673 20729773

[10] Gourévitch B Edeline JM Occelli F Eggermont JJ. Is the din really harmless? Long-term effects of non-traumatic noise on the adult auditory system. Nat Rev Neurosci. (2014) 15:483–91. 10.1038/nrn3744 24946762

[11] Papathanasiou ES Murofushi T Akin FW Colebatch JG. International guidelines for the clinical application of cervical vestibular evoked myogenic potentials: an expert consensus report. Clin Neurophysiol. (2014) 125:658–66. 10.1016/j.clinph.2013.11.042 24513390

[12] Felipe L Santos MA Gonçalves DU. Vestibular evoked myogenic potential (Vemp): evaluation of responses in normal subjects. Pro Fono. (2008) 20:249–54. 10.1590/S0104-56872008000400008 19142468

[13] Chou CH Hsu WC Young YH. Ocular vestibular-evoked myogenic potentials via bone-conducted vibration in children. Clin Neurophysiol. (2012) 123:1880–5. 10.1016/j.clinph.2012.02.059 22386319

[14] Xu XD Zhang XT Zhang Q Hu J Chen YF Xu M. Ocular and cervical vestibular-evoked myogenic potentials in children with cochlear implant. Clin Neurophysiol. (2015) 126:1624–31. 10.1016/j.clinph.2014.10.216 25511635

[15] Wang SJ Chen CN. Hsieh WS, Young YH. Development of vestibular evoked myogenic potentials in preterm neonates. Audiol Neurootol. (2008) 13:145–52. 10.1159/000112422 18087148

[16] Felipe L Kingma H. Ocular vestibular evoked myogenic potentials. Int Arch Otorhinolaryngol. (2014) 18:77–9. 10.1055/s-0034-1388825 25992068 PMC4296944

[17] Strömberg AK Olofsson Å Westin M Duan M Stenfelt S. Changes in cochlear function related to acoustic stimulation of cervical vestibular evoked myogenic potential stimulation. Hear Res. (2016) 340:43–9. 10.1016/j.heares.2015.12.022 26724755

[18] Mattingly JK Portnuff CD Hondorp BM Cass SP. Sudden bilateral hearing loss after cervical and ocular vestibular evoked myogenic potentials. Otol Neurotol. (2015) 36:961–4. 10.1097/MAO.0000000000000764 25853612

[19] Krause E Mayerhofer A Gürkov R Drexl M Braun T Olzowy B. Effects of acoustic stimuli used for vestibular evoked myogenic potential studies on the cochlear function. Otol Neurotol. (2013) 34:1186–92. 10.1097/MAO.0b013e31829ce7b4 23921920

[20] Rodriguez AI Thomas MLA Fitzpatrick D Janky KL. Effects of high sound exposure during air-conducted vestibular evoked myogenic potential testing in children and young adults. Ear Hear. (2018) 39:269–77. 10.1097/AUD.0000000000000484 29466264 PMC5826614

[21] Shin JE Kim CH Park HJ. Influence of thresholds on amplitudes in vestibular evoked myogenic potentials. Auris Nasus Larynx. (2013) 40:352–5. 10.1016/j.anl.2012.10.007 23238175

[22] Hsu YS Wang SJ Young YH. Ocular vestibular-evoked myogenic potentials in children using air conducted sound stimulation. Clin Neurophysiol. (2009) 120:1381–5. 10.1016/j.clinph.2009.04.009 19443267

[23] Sheykholeslami K Megerian CA Arnold JE Kaga K. Vestibular-evoked myogenic potentials in infancy and early childhood. Laryngoscope. (2005) 115:1440–44. 10.1097/01.mlg.0000167976.58724.22 16094120

[24] Pereira AB Silva GS Assunção AR Atherino CC Volpe FM Felipe L. Cervical vestibular evoked myogenic potentials in children. Braz J Otorhinolaryngol. (2015) 81:358–62. 10.1016/j.bjorl.2014.08.019 26163229 PMC9442738

[25] Picciotti PM Fiorita A Di Nardo W Calò L Scarano E Paludetti G. Vestibular evoked myogenic potentials in children. Int J Pediatr Otorhinolaryngol. (2007) 71:29–33. 10.1016/j.ijporl.2006.08.021 16996145

[26] Kujawa SG Liberman MC. Acceleration of age-related hearing loss by early noise exposure: evidence of a misspent youth. J Neurosci. (2006) 26:2115–23. 10.1523/JNEUROSCI.4985-05.2006 16481444 PMC1855187

[27] Thomas MLA Fitzpatrick D McCreery R Janky KL. Big stimulus, little ears: safety in administering vestibular-evoked myogenic potentials in children. J Am Acad Audiol. (2017) 28:395–403. 10.3766/jaaa.15097 28534730 PMC5443117

[28] Portnuff CDF Kleindienst S Bogle JM. Safe use of acoustic vestibular-evoked myogenic potential stimuli: protocol and patient-specific considerations. J Am Acad Audiol. (2017) 28:708–17. 10.3766/jaaa.16071 28906242

